# Lentiviral expression of wild-type *LAMA3A* restores cell adhesion in airway basal cells from children with epidermolysis bullosa

**DOI:** 10.1016/j.ymthe.2024.02.032

**Published:** 2024-02-29

**Authors:** Chun Hang Lau, Maral J. Rouhani, Elizabeth F. Maughan, Jessica C. Orr, Krishna K. Kolluri, David R. Pearce, Elizabeth K. Haughey, Liam Sutton, Sam Flatau, Pablo Lopez Balboa, Maria Laura Bageta, Christopher O’Callaghan, Claire M. Smith, Sam M. Janes, Richard Hewitt, Gabriela Petrof, Anna E. Martinez, John A. McGrath, Colin R. Butler, Robert E. Hynds

**Affiliations:** 1 Epithelial Cell Biology in ENT Research (EpiCENTR) Group, UCL Great Ormond Street Institute of Child Health, University College London, 20c Guilford Street, London WC1N 1DZ, UK; 2 Lungs for Living Research Centre, UCL Respiratory, Division of Medicine, University College London, 5 University Street, London WC1E 6JF, UK; 3 Ear, Nose, and Throat Department, Great Ormond Street Hospital NHS Foundation Trust, London WC1N 3JH, UK; 4 UCL Cancer Institute, University College London, 72 Huntley Street, London WC1E 6DD, UK; 5 Infection, Immunity, and Inflammation Department, UCL Great Ormond Street Institute of Child Health, University College London, 30 Guilford Street, London WC1N 1EH, UK; 6 Department of Dermatology, Great Ormond Street Hospital NHS Foundation Trust, London WC1N 3JH, UK; 7 St John’s Institute of Dermatology, School of Basic and Medical Biosciences, King’s College London, Guy’s Hospital, St Thomas Street, London SE1 9RT, UK

**Keywords:** epidermolysis bullosa, airway basal cells, *in vitro* cell culture, lentiviral transduction, cell adhesion, laminin-332, extracellular matrix, larynx, trachea

## Abstract

The hallmark of epidermolysis bullosa (EB) is fragile attachment of epithelia due to genetic variants in cell adhesion genes. We describe 16 EB patients treated in the ear, nose, and throat department of a tertiary pediatric hospital linked to the United Kingdom’s national EB unit between 1992 and 2023. Patients suffered a high degree of morbidity and mortality from laryngotracheal stenosis. Variants in laminin subunit alpha-3 (*LAMA3*) were found in 10/15 patients where genotype was available. *LAMA3* encodes a subunit of the laminin-332 heterotrimeric extracellular matrix protein complex and is expressed by airway epithelial basal stem cells. We investigated the benefit of restoring wild-type *LAMA3* expression in primary EB patient-derived basal cell cultures. EB basal cells demonstrated weak adhesion to cell culture substrates, but could otherwise be expanded similarly to non-EB basal cells. *In vitro* lentiviral overexpression of *LAMA3A* in EB basal cells enabled them to differentiate in air-liquid interface cultures, producing cilia with normal ciliary beat frequency. Moreover, transduction restored cell adhesion to levels comparable to a non-EB donor culture. These data provide proof of concept for a combined cell and gene therapy approach to treat airway disease in *LAMA3*-affected EB.

## Graphical abstract

**Figure:**
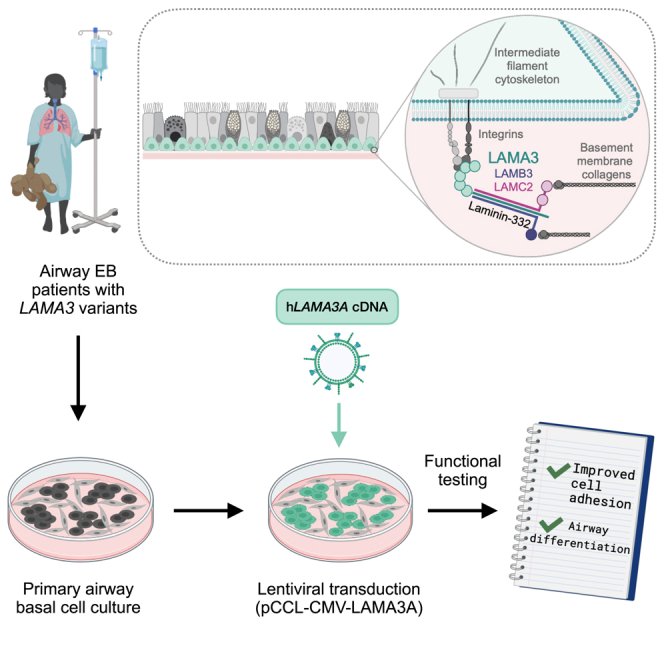

## Teaser

Lau and colleagues demonstrate the prevalence of *LAMA3* variants in epidermolysis bullosa (EB) patients with airway disease. They develop a lentiviral vector to overexpress wild-type *LAMA3* in primary EB patient airway basal cells. These data suggest the potential of combined airway cell and gene therapy for EB patients.

## Introduction

Epidermolysis bullosa (EB) is a group of rare genetic disorders, characterized by extreme fragility and overt blistering of epithelial tissues.^1^ EB manifests most commonly in the skin but can affect diverse epithelial tissues, including those in the respiratory, gastrointestinal, or genitourinary tracts. In common between affected epithelial tissues is compromised cell adhesion, which results in extremely fragile tissue and subsequent blistering and scarring after trivial or mild trauma.^2^ Although life expectancy varies depending on disease phenotype, most forms of EB are associated with severe morbidity.^3^

In airways, EB can present with supraglottic or glottic stenosis secondary to blistering and scarring of the fragile mucosa, which can arise spontaneously or can be induced by minor traumas, such as those caused by coughing or crying. EB is subclassified into four types based on the level of cleavage within the epithelial-subepithelial junction: EB simplex (EBS), junctional EB (JEB), dystrophic EB (DEB), and Kindler EB.^4^ Each type is associated with different causative variants and clinical phenotypes; there are at least 30 clinically distinct phenotypes of EB.^1^ Airway involvement is predominantly observed in two JEB subtypes: JEB severe (JEB-S) and laryngo-onycho-cutaneous syndrome (LOC syndrome).^5^^,^^6^ JEB-S is associated with variants in genes that encode the laminin-332 heterotrimeric protein complex (laminin subunit alpha-3 [*LAMA3*], laminin subunit beta-3 [*LAMB3*], and laminin subunit gamma-2 [*LAMC2*], respectively), a basement membrane extracellular matrix protein,^7^ and, more rarely, with variants in type XVII collagen (*COL17A1*) or integrin subunits alpha 6 (ITGA6), beta 4 (ITGB4), or alpha 3 (ITGA3).^8^^,^^9^ Laminin-332 anchors basal epithelial cells to the basement membrane by connecting integrins on the epithelial cell surface to structural proteins, such as collagens, in the basement membrane. LOC syndrome typically arises from *LAMA3* variants affecting the *LAMA3A* isoform (exons 39–76).^10^^,^^11^ Both JEB-S and LOC syndrome are associated with a high risk of infant mortality,^10^ often due to laryngotracheal complications and airway obstruction.^5^^,^^12^ Notably, JEB intermediate (JEB-I) can also present with laryngeal involvement, but typically with less severe phenotypes due to heterozygosity of the causative pathogenic variants.^13^

The airway management of EB patients involves repeated microlaryngobronchoscopy (MLB) and endoscopic intervention to address the airway stenosis with cold steel instrumentation and/or balloon dilatation. Procedures must be performed delicately to prevent further damage to the mucosa,^6^ and multiple procedures are often required to maintain airway patency. Since patients with laryngeal involvement suffer from severe, life-limiting phenotypes, patients typically present younger than 2 years of age.^5^ In some cases, tracheostomy must be considered. However, the blistering and consequent infections of the fragile skin around the stoma and the dressing can be problematic and the optimal timing of tracheostomy in this patient group to avoid treatment-associated morbidity is not yet clear.

Overall, patients with airway involvement in epidermolysis bullosa are currently underserved by investigative research. In 2020, there were 23 interventional EB clinical trials open internationally, none of which aimed to address airway manifestations of the disease. Here, we describe and characterize a cohort of EB patients referred to the Ear, Nose, and Throat (ENT) Department at Great Ormond Street Hospital for Children (London, UK) between 1992 and 2023. With a view to developing a combined cell and gene therapy for this patient group, we expand patient airway epithelial cells in primary cell culture, generate a lentivirus to overexpress wild-type *LAMA3A* and transduce patient cells *in vitro,* leading to an improvement in *in vitro* cell adhesion.

## Results

### An airway EB clinical cohort

**Figure 1:**
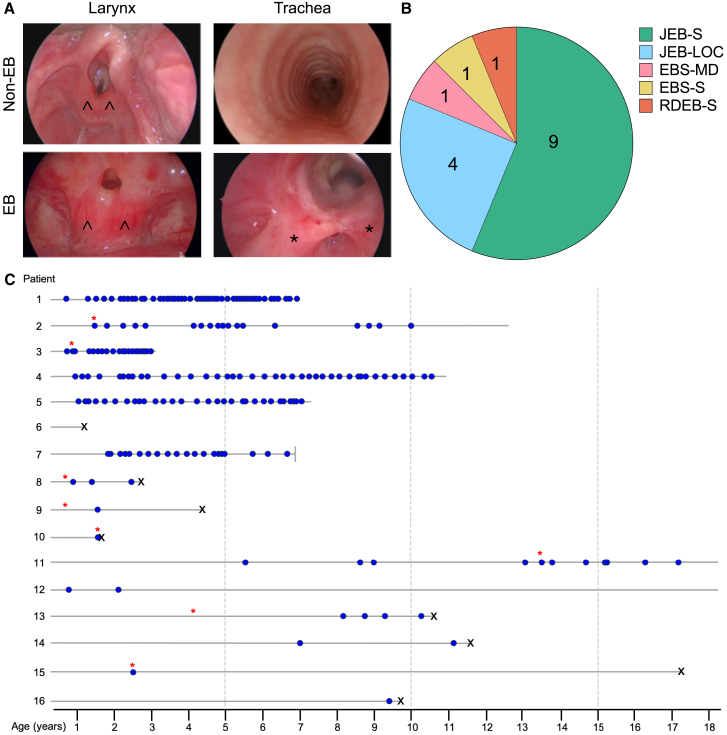
Airway manifestations of EB occur within multiple EB subtypes and are associated with a severe burden of disease (A) Representative clinical photographs from airway endoscopy show severe stenotic scarring throughout the laryngotracheal tree of affected EB patients but not non-EB control patients. Arrowheads indicate the position of the arytenoid cartilages underlying the laryngeal mucosa; in the EB patient, there is accumulation of scar tissue within the laryngeal inlet. Asterisks indicate the bases of a tight band of horizontal scar tissue across the mid-tracheal lumen at the site of previous mild trauma caused by the tip of a tracheostomy tube. (B) Pie chart showing the distribution of EB subtypes within the clinical cohort (n = 16 patients). (C) Longitudinal follow-up of 16 EB patients requiring treatment for airway disease (blue circles denote an endoscopic procedure under general anesthesia, red asterisks denote the insertion of tracheostomy, and black Xs denote the age at death). Patient 7 emigrated from the United Kingdom and was lost to follow-up at the time point indicated by –|. Patients 1, 2, 4, and 5 provided samples for *in vitro* characterization. Patient 6 did not receive MLB procedures at GOSH because the family opted for nonoperative palliative care.

A total of 16 patients (8 male, 8 female) with a diagnosis of EB were referred to the Great Ormond Street ENT team with a median age at referral of 9 months (range 3–116 months); 15 patients were found to have laryngotracheal involvement and 1 patient had tracheal disease only (Figure 1A). The most common phenotypic subtype was JEB-S (9 patients), followed by JEB-LOC (4 patients), EBS with muscular dystrophy (1 patient), severe EBS (1 patient) and severe recessive DEB (1 patient) (Figure 1B). Complete longitudinal follow-up (from referral to current status [alive, dead, or transitioned to adult services]) was available in 15/16 cases and ranged in length from 10 months to 16.5 years. Several patients required numerous MLB procedures to maintain a patent airway (Figure 1C). Half of the cohort (8/16 patients) underwent tracheostomy insertion for ongoing management of their laryngotracheal disease. Eight patients had died at the time of writing, five of whom had previously undergone tracheostomy for their airway disease.

**Figure 2:**
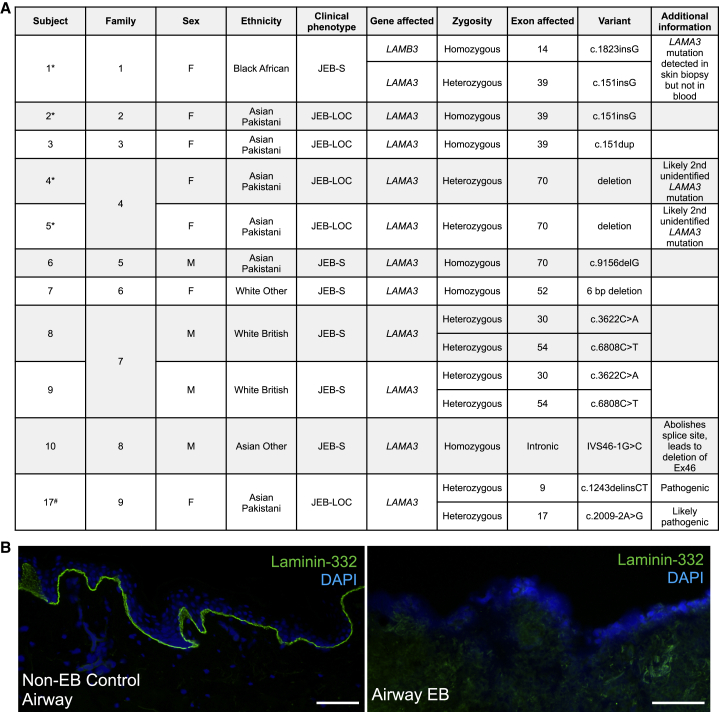
Airway manifestations of EB frequently involve variants of *LAMA3* (A) Table of pathogenic variants and phenotypic subtypes of *LAMA3*-associated EB patients requiring treatment for airway disease. Patients with an asterisk provided samples for *in vitro* characterization. Patient 17 was not managed under ENT services at GOSH, but has contributed research samples. (B) Immunofluorescence staining using an antibody against the mature laminin-332 heterotrimer (laminin-332, green; DAPI, blue) in a tracheal biopsy from a non-EB control patient (left) and a patient with a diagnosis of JEB-LOC due to loss of *LAMA3* function (patient 17; right). Scale bars, 100 μm.

Genotyping data were available for 15/16 patients in the cohort; 10/15 patients had at least 1 *LAMA3* pathogenic variant; 7 patients had pathogenic variants in *LAMA3* affecting both alleles (five homozygous, two double heterozygous) and two patients had a heterozygous *LAMA3* pathogenic variant (we hypothesize that they likely have a further unidentified *LAMA3* variant) (Figure 2A). Patient EB1 had a homozygous *LAMB3* variant that was detected in peripheral blood mononuclear cells, as well as a skin biopsy. A further heterozygous *LAMA3* variant was detected in the skin biopsy. Based on this finding, alongside the markedly decreased LAMA3 protein expression that was observed in cultured tracheal basal cells from EB1, we included this patient within the *LAMA3*-affected group. The remaining 5 patients had pathogenic variants in *LAMB3* (n = 2, homozygous), keratin 14 (*KRT14*; n = 1, heterozygous), collagen alpha-1(VII) chain (n = 1, homozygous), or plectin (n = 1, heterozygous). In another patient with airway EB involvement who was not managed under ENT services at Great Ormond Street Hospital for Children (GOSH) but contributed a research histological sample (patient 17, Figure 2A; JEB-LOC, with *LAMA3* variants), we observed severe loss of reactivity at the tracheal basement membrane to an antibody raised against the mature laminin-332 heterotrimer (Figure 2B).

### EB patient airway epithelial cell culture

**Figure 3:**
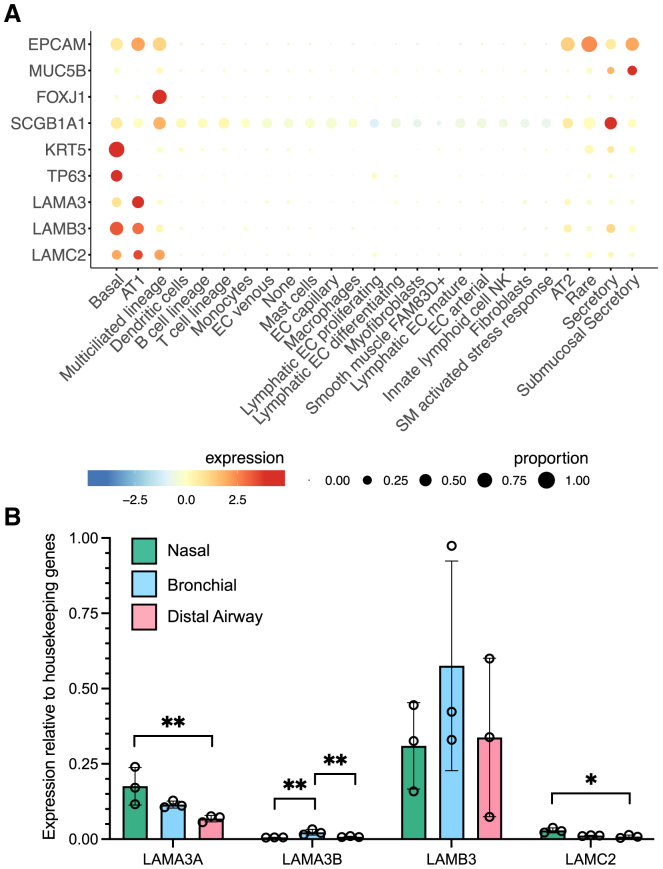
Expression of *LAMA3* in airway basal cells *in vivo* and in primary cell cultures from EB patients (A) Expression of selected airway epithelial cell marker genes (epithelial cell adhesion molecule [*EPCAM*], epithelial cells; mucin 5B [*MUC5B*], mucosecretory cells; forkhead box J1 [*FOXJ1*], multiciliated cells; secretoglobin family 1A member 1 [*SCGB1A1*], club cells; keratin 5 [*KRT5*] and tumor protein p63 [*TP63*], basal cells), *LAMA3*, *LAMB3*, and *LAMC2* in single-cell RNA sequencing data from the integrated HLCA normal dataset.^14^ (B) qPCR analysis of *LAMA3A*, *LAMA3B*, *LAMB3*, and *LAMC2* expression in non-EB adult airway basal cell cultures derived from nasal, airway, or distal airway epithelium (n = 3 donors per condition). Mean expression is shown relative to the average of 2 housekeeping genes (*GAPDH* and *RPS13*). Error bars represent SD; ∗p < 0.05 and ∗∗p < 0.01 in 1-way ANOVAs conducted per gene on ΔCt values.

Interrogation of single-cell RNA sequencing data from the integrated Human Lung Cell Atlas (HLCA) dataset^14^ demonstrated that *LAMA3*, *LAMB3*, and *LAMC2* transcripts are predominantly expressed in airway basal cells and lung alveolar type 1 cells (Figures 3A; S1). *LAMA3* has two major transcript variants that result in two isoforms: A and B. The A isoform consists of exons 39–76 and the B isoform consists of all exons except exon 39. In cultured primary non-EB adult airway basal cells, we found that *LAMB3* was the most highly expressed of the genes encoding laminin-332 components (Figure 3B) and that this was accompanied by higher expression of *LAMA3A* than *LAMA3B* (Figure 3B). Some differences in expression were noted depending on the airway site from which basal cells were isolated, with a higher level of *LAMA3A* expression in proximal than distal airway basal cells (Figure 3B). Combined with the reported success of combined epidermal cell and gene therapy using a γ-retroviral vector carrying *LAMB3* cDNA for patients with JEB,^15^^,^^16^^,^^17^^,^^18^ these data led us to investigate the expansion of primary human airway epithelial cells from patients with EB, with a view to developing a therapy to express the wild-type *LAMA3* gene in the airway epithelium.

**Figure 4:**
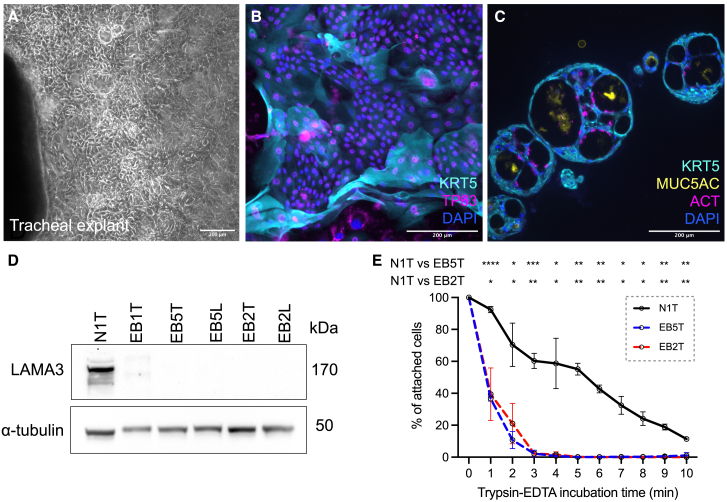
Airway basal cells from EB patients retain stem cell characteristics, lack LAMA3 expression, and are deficient in cell adhesion in cell culture (A) Bright-field microscopy image showing outgrowth of EB patient airway epithelial cells from biopsy tissue in primary cell culture. Scale bar, 200 μm. (B) Immunofluorescence staining of EB tracheal epithelial cells (passage 6) in coculture with 3T3-J2 feeder cells. Cells were stained with antibodies against basal cell markers KRT5 (cyan) and TP63 (magenta). Scale bar, 200 μm. (C) Immunofluorescence staining of EB tracheal epithelial cells following 21 days in 3D tracheosphere culture (assay performed at passage 6). Cells were stained with antibodies against KRT5 (cyan), mucin-5AC (MUC5AC; yellow), and acetylated tubulin (ACT; magenta). Scale bar, 200 μm. (D) Western blot analysis of LAMA3 protein expression in a control tracheal basal cell culture (N1T, passage 5), 3 EB tracheal basal cell cultures (EB1T, EB5T, and EB2T, passage 5), and 2 EB laryngeal basal cell cultures (EB5L and EB2L, passage 5). Alpha tubulin is shown as a loading control. (E) Results from a passive trypsinization assay comparing the trypsin sensitivity of a control tracheal basal cell culture (N1T, passage 5) and 2 EB tracheal basal cell cultures (EB5T and EB2T, passage 5); n = 3 per donor; error bars represent mean ± SD; 2-way ANOVA, ∗p < 0.05; ∗∗p < 0.01; ∗∗∗p < 0.001; and ∗∗∗∗p < 0.0001.

Following informed parental consent, we obtained single, ∼2 mm^2^ matched laryngeal and tracheal pinch biopsies from areas of arytenoid and lateral mid-tracheal mucosa from five children—one pediatric, sex-matched non-EB donor undergoing airway endoscopy for an unrelated reason and four children with known *LAMA3* variants who were undergoing planned endoscopic procedures to manage their airway EB disease (Figure 2A). We successfully established airway epithelial cell cultures from 4/4 tracheal samples and 4/4 laryngeal samples from EB patients by explant culture (Figures 4A–4C). In two-dimensional (2D) culture, epithelial cells expressed KRT5 and tumor protein p63 (TP63; Figure 4B), as expected for airway basal stem cells.^19^^,^^20^ Moreover, EB patient airway epithelial cell cultures were capable of differentiation to both ciliated and mucosecretory cell types in 3D tracheosphere assays (Figure 4C). LAMA3 protein abundance was severely reduced in EB patient basal cell cultures compared to a non-EB control culture (Figure 4D). Functionally, we observed cell adhesion defects in the EB patient-derived cell cultures, with cells proving highly trypsin sensitive compared to the non-EB control cell culture (Figure 4E).

### Lentiviral transduction restores LAMA3 expression in EB airway basal cells

**Figure 5:**
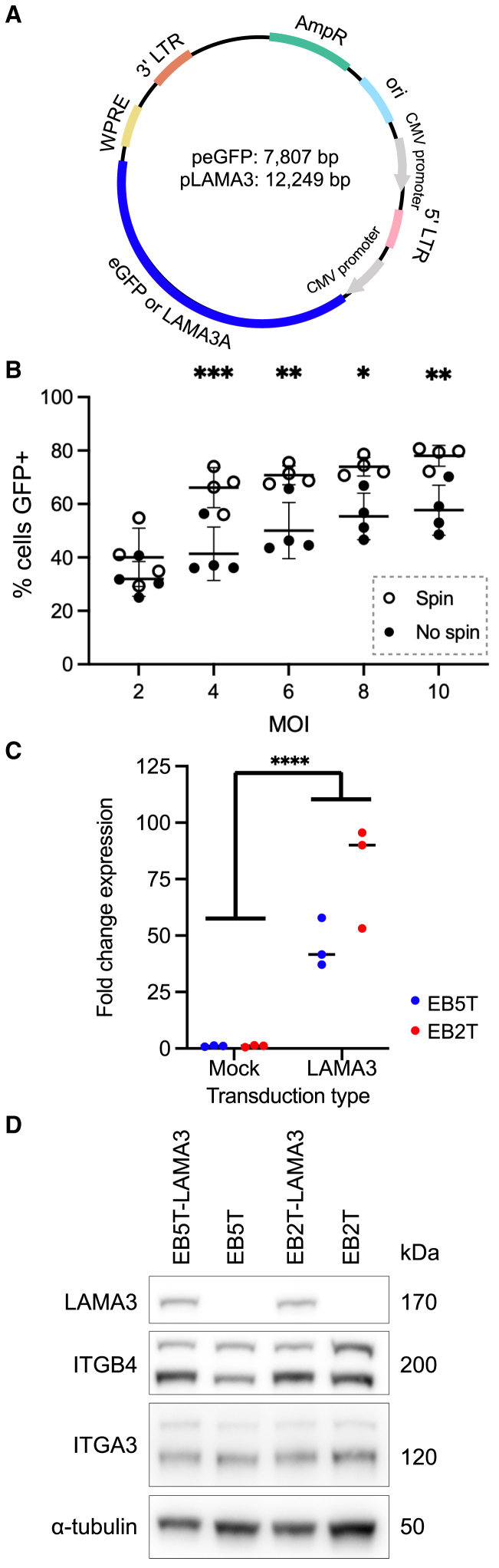
Generation and optimization of a lentiviral vector to express wild-type *LAMA3A* in EB tracheal basal cells (A) Plasmid map showing the 2 lentiviral constructs developed, using identical backbones, with either *EGFP* or *LAMA3A* inserts. (B) Optimization of transduction efficiency in primary human airway basal cells using the EGFP reporter vector. Comparison is between a spin transduction protocol and a nonspin transduction protocol (see materials and methods; n = 4; 2-way ANOVA; ∗p < 0.05, ∗∗p < 0.01, and ∗∗∗p < 0.001). Error bars represent mean ± SD. (C) qPCR analysis of *LAMA3* expression in EB tracheal basal cell cultures from 2 patients (EB5T and EB2T, passage 6). Points represent independent transductions or protocol matched, mock-transduced control cell cultures (n = 3, line indicates median point, 2-way ANOVA; ∗∗∗∗p < 0.0001 in comparison of mock-transduced versus *LAMA3*-transduced groups). (D) Representative western blot analysis of LAMA3, ITGB4, and ITGA3 in 2 *LAMA3*-transduced EB tracheal basal cell cultures (EB5T and EB2T, passage 5 or 6) and matched mock-transduced control cell cultures. Alpha tubulin is shown as a loading control. Blots are representative of 3 independent experiments.

To move toward a combined airway cell and gene therapy for EB patients, we next designed lentiviral vectors that encode either *EGFP* or *LAMA3A* under the control of a constitutive cytomegalovirus (CMV) promoter sequence (Figure 5A). Using the EGFP lentivirus as a reporter system, we optimized viral transduction of non-EB primary human tracheal epithelial cells using a spin transduction method (see materials and methods), achieving transduction efficiencies of >70% at an MOI of 8 (Figure 5B). The colony-forming ability of EGFP transduced basal cells fluorescence-activated cell sorting (FACS) sorted for EGFP positivity did not differ significantly from donor- and passage-matched mock-transduced basal cells (Figure S2A).

**Figure 6:**
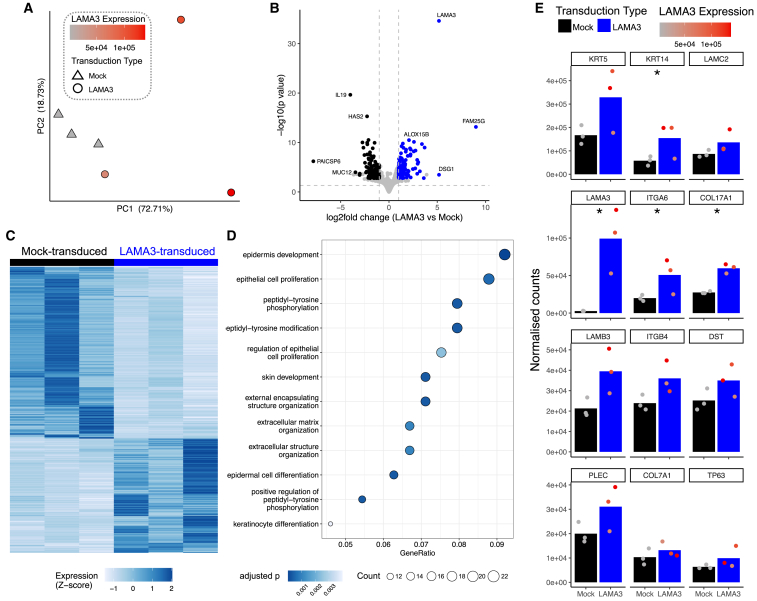
RNA sequencing analysis of *LAMA3*-transduced EB5T tracheal basal cells (A) PCA plot. Shape indicates sample group (triangle, mock-transduced; circle, *LAMA3-*transduced). Individual points are colored by the *LAMA3* expression in that sample. (B) Volcano plot showing the 373 differentially expressed genes between mock-transduced and *LAMA3*-transduced samples. Genes that are upregulated in *LAMA3*-transduced cells are colored blue, and those that are downregulated are black. Labeled genes are annotated manually (a full list of differentially expressed genes can be found in Table S1). (C) Heatmap showing the 373 differentially expressed genes between mock-transduced and *LAMA3*-transduced samples. Expression for each gene is scaled. (D) Gene set enrichment analysis of the differentially expressed genes. (E) Barplots showing the expression (normalized counts) of genes associated with hemidesmosomes. Bars represent the mean (n = 3). Individual expression values are represented as points, which are colored according to the *LAMA3* expression in that sample. The asterisk indicates significant adjusted p values.

Next, we transduced EB patient basal cells with the *LAMA3* lentivirus using the optimized spin transduction protocol. Increased *LAMA3* mRNA abundance (EB5T, mean = 45.5-fold increase; EB2T, mean = 79.6-fold increase; Figure 5C) and protein abundance were seen in two independent EB patient basal cell cultures with distinct causative LAMA3 mutations following transduction (Figure 5D). Expression of ITGB4 and ITGA3, however, remained unaltered (Figure 5D). The expression of *LAMA3* mRNA (EB5T, mean = 3.3-fold increase; EB2T, mean = 8.4-fold increase; Figure S2B) and protein (Figure S2C) in these cultures was higher than in the non-EB control cultures, consistent with its constitutive expression being driven by the strong CMV promoter. To understand the overall impact that overexpression of wild-type *LAMA3* had on EB airway basal cells, we performed RNA sequencing on three independent transductions of tracheal basal cells from EB5 as well as nontransduced cells. Principal-component analysis (PCA) showed clustering based on transduction status, with more variability between *LAMA3*-transduced cultures than mock-transduced cultures (Figure 6A). We identified a total of 373 differentially expressed transcripts, with 149 upregulated in *LAMA3*-transduced cultures (padj <0.05, log2 fold change >1) and 224 downregulated (padj <0.05, log2 fold change <−1; Figures 6B and 6C; Table S1). Of the most significant differentially expressed genes, *IL19* (p = 1.40 × 10^−16^) and *HAS2* (p = 2.42 × 10^−12^) were notable for being downregulated after *LAMA3* transduction and having previously been associated with inflammatory pathogenesis involving the airway epithelium.^21^^,^^22^ Gene set enrichment analysis suggested that overexpression of wild-type *LAMA3* was associated with more widespread changes in epithelial cell phenotype with significant enrichment of keratinocyte differentiation, epithelial cell proliferation, and extracellular matrix organization (Figure 6D). Indeed, analysis of genes that are associated with cell adhesion via hemidesmosomes indicated a general trend toward upregulation of these genes in *LAMA3*-transduced cultures, with significant upregulation observed for *LAMA3* (p = 2.35 × 10^−31^), *KRT14* (p = 0.024), *ITGA6* (p = 0.018), and *COL17A1* (p = 8.79 × 10^−5^; Figure 6E).

**Figure 7:**
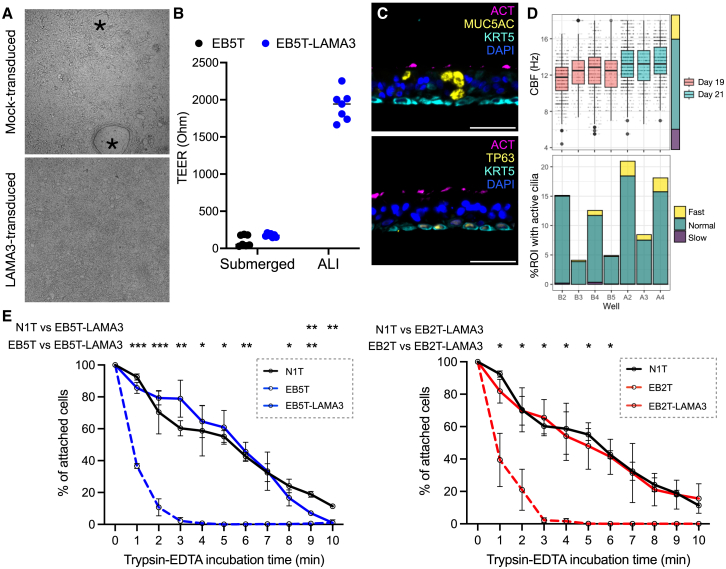
*LAMA3* transduction restores the ability of EB tracheal basal cells to differentiate in ALI cultures and reverses *in vitro* cell adhesion defects (A) Bright-field microscopy images showing mock-transduced (EB5T) and *LAMA3*-transduced (EB5T-LAMA3) 72 h after air lift. Holes within the epithelium are indicated by black asterisks. (B) TEER values for submerged basal cell cultures 24 h after seeding (i.e., submerged cultures before air lift) and after 19–21 days at an ALI. n = 7 wells of EB5T cells from 2 independent experiments. No values are shown for nontransduced cells at ALI because 7/7 wells failed. (C) Immunofluorescence staining for basal cells (KRT5, cyan), mucosecretory cells (MUC5AC, yellow), and multiciliated cells (ACT, magenta; top) or basal cells (KRT5, cyan; TP63, yellow) and multiciliated cells (ACT, magenta; bottom) in EB5T-LAMA3 ALI cultures. Scale bars, 50 μm. (D) High-speed video microscopy analysis of CBF in 7 EB5T-LAMA3 ALI culture wells from 2 independent experiments. Top:plot of CBF (Hz; box represents the interquartile range, whiskers represent the range); bottom: plot of the distribution of ciliary beat speed within ROIs in each well replicate. (E) Passive trypsinization assay comparing the trypsin sensitivity of a control tracheal basal cell culture (N1T, passage 5), the EB5T (left, passage 5) and EB2T (right, passage 5) LAMA3-transduced EB tracheal basal cell cultures, and matched mock-transduced cultures (n = 3; error bars represent mean ± SD; 2-way ANOVA; ∗p < 0.05, ∗∗p < 0.01, and ∗∗∗p < 0.001). Data from the N1T control cells and the mock-transduced EB5T and EB2T cells are also shown in Figure 4E.

In colony-formation assays, *LAMA3*-transduced cells behaved similarly to mock-transduced donor- and passage-matched cells (Figure S2D). In air-liquid interface (ALI) assays, mock-transduced EB cultures failed within 5–7 days of the introduction of an air interface, with holes forming within the epithelium (Figure 7A). Transepithelial electrical resistance (TEER) measurements indicated minimal differences between mock-transduced and *LAMA3*-transduced cells in submerged culture (Figure 7B). TEER values for established ALI cultures of *LAMA3*-transduced cells were within the normal range for non-EB cells (i.e., >250 Ω; Figure 7B).^23^ *LAMA3*-transduced ALI cultures contained multiciliated cells (Figure 7C), and the ciliary beat frequency (CBF) of these was within the expected range for non-EB control cultures (normal range = 7–16 Hz; Figure 7D).^23^ Importantly, the *in vitro* cell adhesion of *LAMA3*-transduced EB patient airway basal cells was comparable to a non-EB donor control cell culture, demonstrating functional correction of the observed cell adhesion defect (Figure 7E).

## Discussion

In this study, we have described a cohort of patients with EB treated by the ENT Department at GOSH. This cohort of 16 children with airway disease represents a minority of all of the children treated for EB at the center, with 425 patients seen in total over a comparable time period. Although airway manifestations within EB are rare, the affected subgroup suffers from high morbidity and an inexorable worsening of both quality of life and life expectancy. Airway EB patients currently have limited and suboptimal therapeutic options; we show that they require multiple and frequent endoscopic airway dilatations under general anesthesia to manage their increasingly stenotic laryngotracheal airways.

Consistent with previous reports,^11^^,^^24^^,^^25^ we associate upper airway manifestations of EB with alterations in the *LAMA3* gene. In mice, loss of *LAMA3* is lethal in the perinatal period as a result of severe defects in epithelial tissues.^26^ Interestingly, loss of *LAMA3* in the mouse lung leads to increased deposition of collagen and inflammation, which may be relevant to airway EB pathogenesis.^27^ Upper airway and tracheal phenotypes have also been observed in dogs with *LAMA3*-mutant EB.^28^ In EB patients, *LAMA3* variants are associated with LOC syndrome, but we also identified non-LOC patients who had *LAMA3* variants and airway symptoms, predominantly from the JEB-S subtype. It is unclear why *LAMA3* variants may confer airway tropism compared to other EB-causative variants, and this suggests an avenue for future fundamental research on laminin-332 subunit biology—for example to compare the relative dependency of different epithelia on laminin-332-containing adhesion complexes versus other complexes.

There has been enthusiasm for gene therapy in monogenic respiratory diseases for several decades, but the delivery of viral vectors to airway epithelium has proved challenging.^29^ The evolution of the epithelium as a physical barrier to inhaled particles means that mucociliary clearance and cell-to-cell adhesion via tight junctions inhibit efficient viral transduction *in vivo*. Laminin-332 localizes to both hemidesmosomes (through interaction with integrin α6β4) and focal adhesions (with integrin α3β1) to mediate cell-matrix adhesion,^30^ but is not involved in cell-to-cell adhesion via tight junctions. As such, the predisposition of the EB airway epithelium for gene transfer remains uncertain, and preclinical preconditioning approaches that expose basal cells before infection (e.g., through the use of polidocanol to remove luminal cell types)^31^ are often unsuitable for clinical application. Although significant progress is being made in *in vivo* lentiviral delivery to overcome these challenges by optimizing pseudotype^32^ and preconditioning approaches,^33^ we believe that many of the issues encountered in other disease settings are relevant for *in vivo* gene delivery in EB airways.^34^

By combining primary airway epithelial cell culture with lentiviral transduction, we provide proof of principle for a combined cell and gene therapy approach for airway EB. One advantage of this approach is the ability to use autologous cells to enable transplantation into a recipient without provoking an immunological response and/or rejection of the graft. Furthermore, a variety of *LAMA3* variants were observed in our cohort (Figure 1C), so lentiviral expression of wild-type *LAMA3* represents a more universally applicable therapy than individualized gene correction. Airway basal cells express the laminin-332 components *in vivo*, suggesting them as a suitable target cell for gene correction. However, the use of a constitutively active promoter is a limitation of the approach described here because *LAMA3* would not be downregulated upon basal cell differentiation and, although we demonstrate that these cells functionally differentiate, the consequences of ectopic LAMA3 expression in multiciliated, mucosecretory, and other airway epithelial subpopulations require further characterization. As such, future work to generate a clinically translatable lentiviral vector should investigate the use of basal cell-specific promoter sequences to restore *LAMA3* expression.

Given the rate of cell proliferation that is observed in the cell culture conditions used in this study, it is highly feasible to expand sufficient numbers of basal cells for transplantation.^23^^,^^35^^,^^36^ It is additionally likely that clinical product generation could be further optimized to minimize both time in culture and time to treatment.^37^ Thus, we anticipate that this method comfortably provides sufficient cell numbers for clinical airway engraftment in this patient group, including the creation of any patient-specific cryopreserved cell stocks that may prove to be necessary for repeated administration.

A major barrier to airway cell therapy in general and for the approach foreseen here has been the lack of surgical methods to deliver epithelial cells to the airways.^38^ Although the transplantation of airway basal cells has been possible in rodent models, the preconditioning methods and routes of delivery used would be unacceptable in patients, and autologous cells are rarely used, with studies often using immunodeficient mice to enable transplantation.^39^ However, several methods under preclinical application, such as endobronchial cell delivery or stent-based transplantation, may enable basal cell delivery to patients. EB represents a strong candidate for clinical translation of an airway cell therapy, given that corrected cells may outcompete recipient epithelial cells *in vivo* as a consequence of enhanced cell adhesion in basal stem cells. This is in contrast to other monogenic lung disorders; for example, cystic fibrosis is caused by pathogenic variants in the cystic fibrosis transmembrane conductance regulator gene, but the gene is not expressed by basal cells. This limits the opportunity for gene-corrected cells to outcompete resident stem cells following transplantation.

Overall, our study provides early proof-of-principle data for a lentivirus-based combined cell and gene therapy for patients with airway manifestations of EB.

## Materials and methods

### Case information collection

A retrospective single-center case series study was performed to identify all EB patients with laryngotracheal involvement who have been managed at GOSH. Patients were identified from a hospital database, and their treatment at GOSH occurred between January 1992 and June 2023. Data were collected on patient demographics, EB type and subtype, genotyping, frequency of airway interventions, need for tracheostomy insertion, and the involvement of other organs. Ethnicity is described according to definitions from the UK Census 2021. Genotyping was performed by the Robin Eady National EB Diagnostic Laboratory (Guy’s Hospital, London, UK).

### Human airway tissue collection

Ethical approval for primary cell culture studies involving human cells was obtained through the Living Airway Biobank at University College London (National Research Ethics Committee [REC] reference 19/NW/0171). Following discussion and informed written consent from their parents/guardians, laryngeal and tracheal luminal biopsies were obtained using cupped microlaryngoscopic biopsy forceps from patients during planned rigid laryngotracheobronchoscopy under general anesthesia (which was necessary as part of their routine clinical care). Additional primary human non-EB adult airway epithelial cell cultures were derived under REC reference 18/SC/0514.

### Primary airway epithelial cell culture

To initiate primary epithelial cell cultures, fresh tracheal or laryngeal biopsies were placed in culture on 3T3-J2 feeder layers and cultured as explants, as previously reported.^23^^,^^40^ Adult nasal basal cells were isolated from brush biopsies by shaking the samples in 15-mL falcon tubes to release epithelial cells before centrifugation at 300 × *g* and cell plating. Adult bronchial and distal airway basal cells were isolated by dissecting airways from lobectomy samples, mincing the tissue with a scalpel, and processing with a previously reported dispase/trypsin digestion method to achieve a single-cell suspension for plating.^41^ Culture initiation was performed in primary epithelial cell culture medium consisting of DMEM (Gibco) and F12 (Gibco) in a 3:1 ratio with 1× penicillin/streptomycin (Gibco) and 5% fetal bovine serum (FBS) (Gibco) supplemented with 5 μM Y-27632 (Cambridge Bioscience), 25 ng/mL hydrocortisone (Sigma-Aldrich), 0.125 ng/mL EGF (Sino Biological), 5 μg/mL insulin (Sigma-Aldrich), 0.1 nM cholera toxin (Sigma-Aldrich), 250 ng/mL amphotericin B (Fisher Scientific), and 10 μg/mL gentamicin (Gibco). 3T3-J2 mouse embryonic fibroblasts (purchased from Kerafast) were cultured in DMEM containing 7.5% bovine calf serum (Cytiva) and 1× penicillin/streptomycin (Gibco). To prepare feeder layers the day before receipt of the biopsies, 3T3-J2 cells were mitotically inactivated by treatment with 4 μg/mL mitomycin C for 3 h. Feeder cells were trypsinized and plated at a density of 20,000 cells/cm^2^.^40^

After 7–10 days, outgrowth of epithelial cells was visible around each biopsy. Cells were then differentially trypsinized and passaged by incubation with 0.05% trypsin/EDTA (Gibco) at room temperature for 2 min to detach feeder cells, which were then removed by aspiration. A second round of trypsinization was performed at 37°C to detach the epithelial cells. Trypsin was neutralized using primary epithelial cell culture medium, cells were centrifuged at 300 × *g* for 5 min, resuspended in fresh primary epithelial cell culture medium, and seeded on a freshly prepared feeder layer. Subsequent passages were performed when cell confluency reached 80%. The 3D tracheospheres were generated using a previously published protocol.^40^

For colony-formation assays, 1,000 cultured human airway epithelial cells were seeded per well of a 6-well plate containing inactivated 3T3-J2 feeder cells. Medium was carefully changed on days 4 and 8 of culture before the experiment was terminated on day 12. Colonies were fixed for 10 min in 4% paraformaldehyde (PFA) in PBS, stained using crystal violet (Sigma-Aldrich) at room temperature for 20 min, and washed repeatedly in water. Colonies, defined as contiguous groups of >10 cells, were counted manually using a light microscope. Colony-forming efficiency was calculated as: (number of colonies formed/number of seeded cells) × 100.

The trypsin sensitivity of EB patient epithelial cells was compared to non-EB donor epithelial cells by seeding 20,000 epithelial cells (obtained by differential trypsinization) per well of a 96-well plate in 3T3-J2 conditioned medium, which was produced as previously described.^42^ The following day, cells were washed once with PBS before trypsin was added to all of the wells. Trypsinization of wells was stopped every 60 s in a subset of wells by the removal of trypsin and the addition of primary epithelial cell culture medium to neutralize the trypsin reaction. At the end of the experiment, all of the wells were washed once with PBS, and the cells were fixed in 100 μL 4% PFA for 20 min at room temperature. After fixation, 40 μL 2.3% crystal violet solution (Sigma-Aldrich) was added to the wells for 15 min at room temperature. The plate was then washed in a Tupperware box under a continuous flow of water and dried overnight on paper towels. A total of 150 μL 10% acetic acid was added to each well to dissolve the crystal violet, and the plate was read at 570 nm absorbance on a plate reader.

ALI cultures were performed as previously described in PneumaCult medium (STEMCELL Technologies).^43^ TEER measurements were made using the EVOM Epithelial Voltohmmeter (World Precision Instruments). Videos for the CBF analysis were obtained on a Nikon Eclipse TiE inverted microscope with a Prime BSI Express camera and a Nikon Super Plan Fluor ELWD 20XC PH objective. For CBF analysis, we captured 512 frames at a frame rate of 188 fps and calculated CBF of 6,400 regions of interest (ROIs) per video using the ciliR package with a background noise level of 2.^44^

### Western blotting

Epithelial cells were collected by differential trypsinization from T25 flasks and resuspended in PBS. Cells were pelleted and resuspended in 120 μL Pierce RIPA lysis buffer (Thermo Fisher Scientific) and 1× protease and phosphatase inhibitor, and then incubated on ice for 15 min. Lysates were spun at 13,800 × *g* at 4°C for 15 min, and the supernatants containing proteins were transferred to a new tube. The protein concentration was determined using a Pierce BCA protein assay kit (Thermo Fisher Scientific). Laemmli SDS sample buffer (Thermo Fisher Scientific) and reducing agent (Thermo Fisher Scientific) were added, and 30 μg of protein was run in a Bis-Tris, 4%–12% gel in Bolt MOPS SDS running buffer at 150 V for 60 min. The protein was transferred to a polyvinylidene fluoride membrane in 25 mM Tris, 192 mM glycine, and 20% methanol at 70 V at 4°C for 2 h. The membrane was blocked with 5% skim milk in Tris-buffered saline containing 0.1% Tween 20 (TBST; Sigma-Aldrich), followed by washing in TBST and incubated in primary antibodies (Table S2) in TBST at 4°C overnight. The membrane was washed in TBST and incubated with secondary antibodies at room temperature for 2 h. After washing with TBST, the membrane was developed using western horseradish peroxidase substrate (Merck) according to the manufacturer’s instructions and imaged using a Bio-Rad ChemiDoc Imager.

### qRT-PCR

Epithelial cells were collected from T25 flasks by differential trypsinization and washed with PBS. RNA was extracted from pellets using the PureLink RNA Mini Kit (Thermo Fisher Scientific), followed by cDNA synthesis using the LunaScript RT Supermix kit (NEB). qPCR was performed against different gene targets (Table S3) using a QuantStudio 5 qPCR machine.

### RNA sequencing and analysis

Processed single-cell RNA sequencing data were obtained from the integrated HLCA project.^14^ A Seurat object containing the “core,” normal HLCA samples was downloaded and visualized using ShinyCell.^45^

For bulk RNA sequencing, RNA was extracted using the PureLink RNA Mini Kit (Thermo Fisher Scientific) as per the manufacturer’s protocol. RNA concentrations were quantified using a Qubit 3.0 Fluorometer (Thermo Fisher Scientific). DNA libraries were constructed using the KAPA mRNA HyperPrep kit (Roche) and sequenced on a NextSeq 2000 on a P2 flow cell (at UCL Genomics), giving a median of 27.3 million (range 21.9–31.6) 56-bp paired-end reads per sample.

FASTQ files were processed using the nf-core/rnaseq pipeline (version 3.11.2) with the GRCh38 reference genome.^46^ Computed gene counts were used for analyses using R (version 4.3.1). PCA and differential expression analyses were performed on output from DEseq2 (version 1.40.2). Ensembl gene IDs were matched to gene symbols using BiomaRt (version 2.56.1). The hemidesmosome gene list was manually curated. Processed data and analysis code are available via Zenodo (https://doi.org/10.5281/zenodo.10696184).

### Immunofluorescence staining

Immunofluorescence staining of EB patient samples was performed at the National Diagnostic Epidermolysis Bullosa Laboratory, Guy’s Hospital (London, UK) using an in-house anti-laminin-332 antibody. Images were acquired using a Zeiss LSM700 confocal microscope.

For immunofluorescence staining of primary epithelial cells, 1.1 × 10^5^ epithelial cells per cm^2^ were seeded in a chamber slide containing feeder cells in primary epithelial cell culture medium. When cells reached 80% confluency, they were fixed in 4% PFA and stored in PBS until staining. Wells were blocked in PBS containing 10% FBS and 0.01% Triton X-100 for 1 h at 4°C. Primary antibody staining was performed overnight at 4°C in PBS containing 10% FBS, wells were washed twice with PBS, and secondary antibody staining was performed by diluting species-appropriate Alexa Fluor secondary antibodies (1:500; Molecular Probes) in PBS containing 10% FBS for 2 h at room temperature. Nuclei were visualized by the addition of DAPI (Sigma-Aldrich) at 0.1 μg/mL in PBS for 20 min. ALI cultures were fixed for 24 h in 10% formalin and then stored in PBS at 4°C until processing. Transwell membranes were excised from the transwell inserts before processing. Tracheospheres were fixed by resuspension in 4% PFA and embedded within in HistoGel specimen processing gel (Thermo Fisher Scientific) before further processing. Samples were processed using a Leica TP 1050 vacuum tissue processor and embedded in paraffin. The 5-μm sections were prepared using a Microm HM 325 microtome, and slides were dewaxed in xylene before antigen retrieval by boiling in 10 mM sodium citrate pH 6 using a microwave. Samples were washed in PBS before a hydrophobic ring was drawn around the sample using an ImmEdge pen (Vector Laboratories). Samples were then blocked in PBS containing 1% BSA (Merck), 5% normal goat serum (Abcam), and 0.1% Triton X-100 for 1 h. Staining with primary antibody (Table S2) was performed in blocking buffer overnight at 4°C. After washing twice in PBS, samples were incubated with species-appropriate Alexa Fluor secondary antibodies (1:1,000; Molecular Probes) in blocking buffer at room temperature for 2 h. Nuclei were visualized by the addition of DAPI (Sigma-Aldrich) at 0.1 μg/mL in PBS for 20 min. Fluorescence images were acquired using a Leica DMi8 microscope and were analyzed in ImageJ2.

### Plasmids

*LAMA3A* cDNA was purchased from Sino Biological and amplified by PCR using LAMA3_F and LAMA3_R (primer sequences are listed in Table S3) to add overlapping sequences. *LAMA3A* was then cloned into the lentiviral plasmid pCCL-CMV-flT vector previously described.^47^ Sequences coding for two new restriction sites, NheI and MluI, were first introduced in between the BamHI and SalI sites within the pCCL-CMV-flT plasmid in place of flT. *LAMA3A* was incorporated into the plasmid using the NheI and MluI restriction sites, creating the *LAMA3* vector designated pCCL-CMV-LAMA3 (‘pLAMA3’). pCCL-CMV-EGFP (‘pEGFP’) was previously described.^47^ All of the sequences were confirmed by Oxford Nanopore whole plasmid sequencing (Full Circle Labs).

### Lentivirus production, cell transduction, and FACS

To generate lentivirus, HEK293T cells were transfected with pMD2.G and pCMVR8.74 (Addgene 12259 and 22036, kind gifts from Prof. Adrian Thrasher, University College London) and pEGFP or pLAMA3 using jetPEI transfection reagent (Polyplus). The virus was collected 48 h posttransfection and concentrated by combining the supernatants with PEGit concentrator (5×; System Biosciences LV810A-1) overnight at 4°C. After centrifugation at 1,500 × *g* for 45 min, the supernatant was removed and the pellet was resuspended in 1/10th of the original volume of PBS. Concentrated supernatants were stored at −80°C until use.

To estimate the virus titer, HEK293T cells were transduced with different volumes of virus, and flow cytometry was used to determine the percentage of transduced cells 2 days posttransduction. For pEGFP transduction, cells were resuspended in PBS and analyzed on a BD LSRII flow cytometer (BD Biosciences).

For transduction, epithelial cells were trypsinized as described above and resuspended in a 1:1 mixture of 3T3-J2 conditioned medium and primary epithelial cell culture medium.^42^ A total of 2 × 10^5^ epithelial cells per well were seeded in a 6-well cell culture plate. Immediately after cell seeding, lentivirus (MOI 8) was added to the cells. The plate was centrifuged at 920 × *g* at 30°C for 1 h, followed by incubation at 37°C and 5% CO_2_. The medium containing virus was changed to fresh medium containing 3T3-J2 feeder cells 3 h posttransduction. For comparison of transduction with and without centrifugation, epithelial cells were seeded and allowed to attach for 3 h before the addition of virus for a further 3 h in the “no spin” control group. Mock-transduced cells were subjected to the same protocol as transduced cells but without the addition of lentivirus. EGFP-transduced epithelial cells were isolated by FACS using a BD LSRII cell analyzer (BD Biosciences) and then further expanded on 3T3-J2 feeder layers as described above.

### Statistical analysis

Chart plotting and statistical analyses were performed in GraphPad Prism (version 9.2.0) or R (version 4.3.1). Error bars represent mean ± SD.

## Data and code availability

Bulk RNA sequencing data associated with this paper (both processed counts and raw data) are publicly available via GEO: GSE249910. Processed data and analysis code are available via Zenodo (https://doi.org/10.5281/zenodo.10696184).

## Supplemental information

Document S1. Figures S1 and S2 and Tables S2 and S3

Table S1. Differentially expressed genes in bulk RNA sequencing experiment comparing EB5T and EB5T-LAMA3 cultured airway basal cells

Document S2. Article plus supplemental information
