# Supplementary material for: Lentiviral expression of wild-type LAMA3A restores cell adhesion in airway basal cells from children with epidermolysis bullosa
Source: Mol Ther. 2024 Feb 29;32(5):1497–509. doi: 10.1016/j.ymthe.2024.02.032 (PMC11081864; doi:10.1016/j.ymthe.2024.02.032)
Supplement: Document S1. Figures S1 and S2 and Tables S2 and S3 [file mmc1.pdf]

YMTHE, Volume *32*

Supplemental Information

Lentiviral expression of wild-type *LAMA3A* restores cell adhesion in airway basal cells from children with epidermolysis bullosa

Chun Hang Lau, Maral J. Rouhani, Elizabeth F. Maughan, Jessica C. Orr, Krishna K. Kolluri, David R. Pearce, Elizabeth K. Haughey, Liam Sutton, Sam Flatau, Pablo Lopez Balboa, Maria Laura Bageta, Christopher O'Callaghan, Claire M. Smith, Sam M. Janes, Richard Hewitt, Gabriela Petrof, Anna E. Martinez, John A. McGrath, Colin R. Butler, and Robert E. Hynds

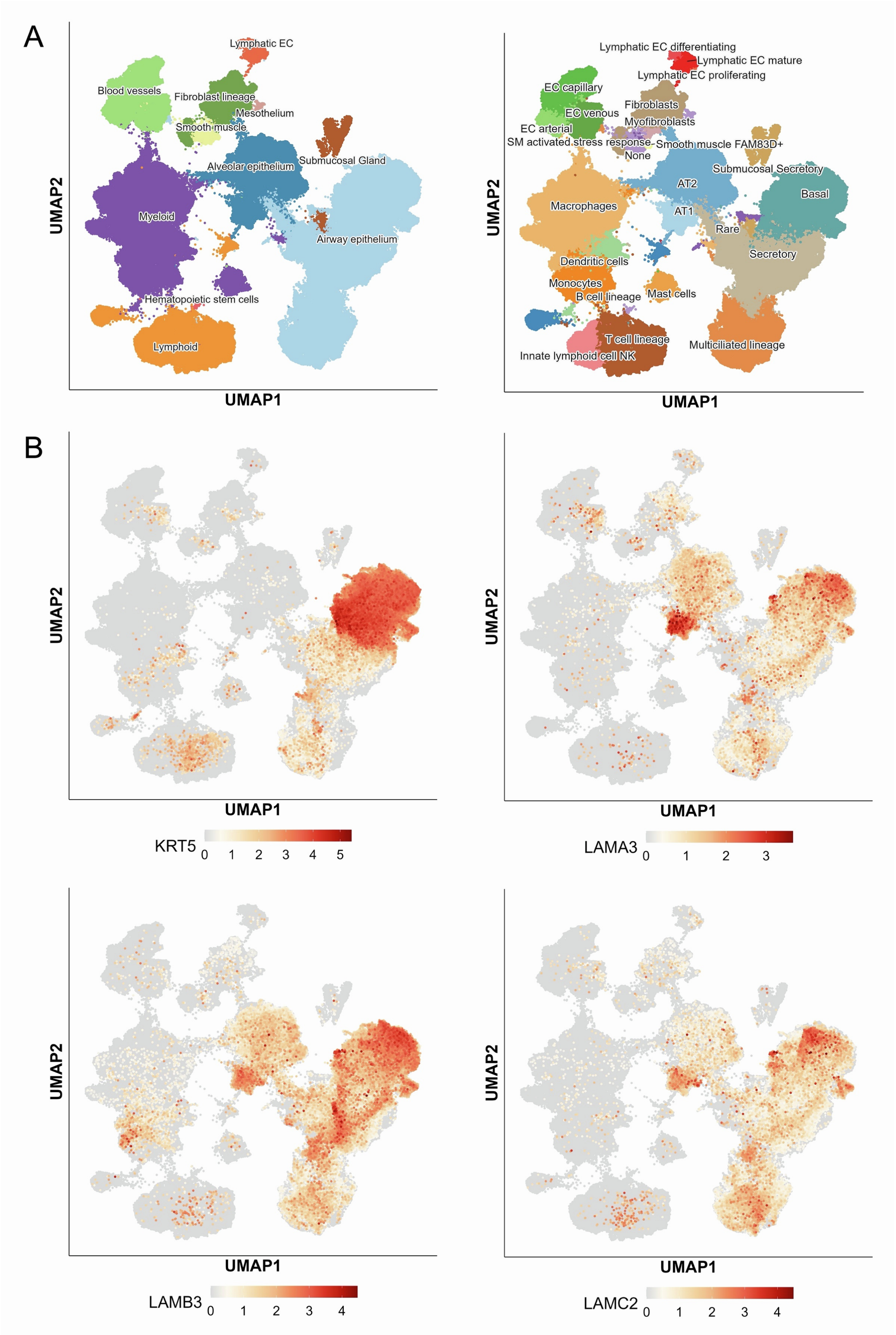

Figure S1: Analysis of laminin subunit alpha-3 (*LAMA3*) expression in the integrated Human Lung Cell Atlas dataset. (**A**) UMAP plots with the annotation of cell types from the Human Lung Cell Atlas at either low (left) or high (right) resolution. (**B**) UMAP plots showing the expression of keratin 5 (*KRT5*; top left), laminin subunit alpha-3 (*LAMA3*; top right), laminin subunit beta-3 (*LAMB3*; bottom left) and laminin subunit gamma-2 (*LAMC2*; bottom right).

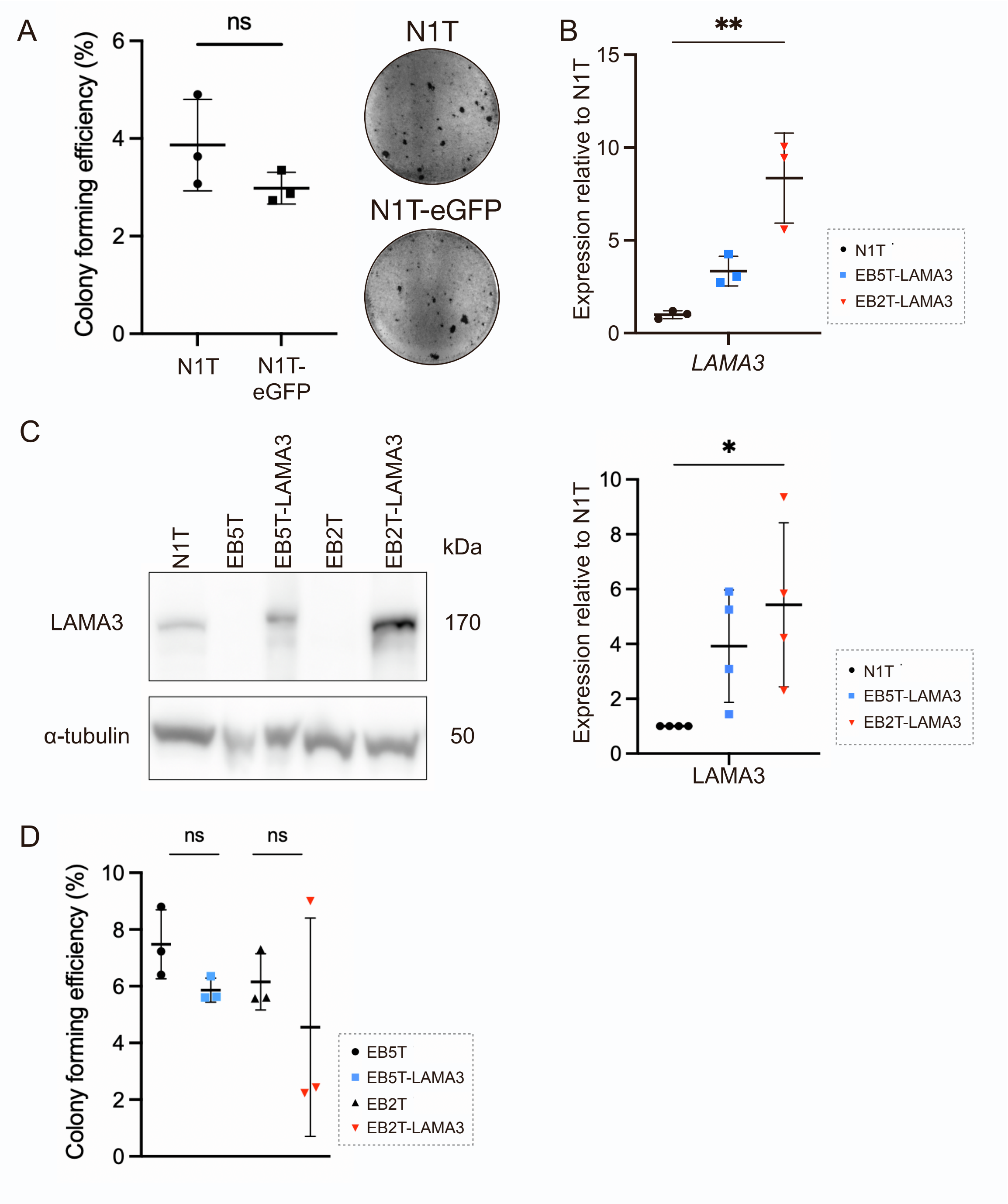

Figure S2: Effects of *eGFP*- or laminin subunit alpha-3 (*LAMA3*)-transduction on cultured primary human airway basal cells. (**A**) Colony formation assay comparing primary non-EB tracheal basal cells transduced with eGFP with mock-transduced control cells (passage 6, n = 3; error bars represent mean ± SD; two-tailed, unpaired t-test; ns = non-significant). Representative well images are shown in insets. (**B**) qPCR analysis of *LAMA3* expression a control tracheal basal cell culture (N1T, passage 6) and two EB tracheal basal cell cultures (EB5T and EB2T, passage 6; n = 3). (**C**) Representative Western blot analysis of LAMA3 expression in a control tracheal basal cell culture (N1T, passage 6) and two *LAMA3*-transduced EB tracheal basal cell cultures (EB5T and EB2T, passage 6; left). Quantification of western blot data (right; n = 4 independent transductions; one-way ANOVA; * denotes p<0.05). (**D**) Colony formation assay comparing two *LAMA3*-transduced EB tracheal basal cell cultures (EB5T and EB2T, passage 5) and matched mock-transduced control cell cultures (n = 3; one-way ANOVA; ns = non-significant).

Table S1: Differentially expressed genes in bulk RNA sequencing experiment comparing EB5T and EB5T-LAMA3 cultured airway basal cells. Information is provided as a separate Excel spreadsheet.

Table S2: Antibodies.

**Antibody**	**Source**	**Catalog Number**	**Species**	**Dilution**
Laminin-332	National Diagnostic EB Laboratory	-		-
KRT5	BioLegend	905901	Chicken	1:500
TP63	Abcam	ab53039	Rabbit	1:200
TP63	Abcam	ab124762	Rabbit	1:300
MUC5AC	Merck	M5293	Mouse	1:500
MUC5AC	Thermo Fisher Scientific	PA5-80832	Rabbit	1:200
Acetylated tubulin (ACT)	Merck	T6793	Mouse	1:500
LAMA3	Bio-Techne	MAB21441	Mouse	1:250
α-tubulin	Cell Signaling Technologies	9099S	Rabbit	1:1000
Integrin β4	Abcam	ab133682	Rabbit	1:1000
Integrin α3	Abcam	ab242196	Mouse	1:1000

Table S3: Primer Sequences.

**Primer name**	**Sequence 5' to 3'**
LAMA3_F	GAGGTCTATATAAGCAGAGCTGGCTAGCCGCCACCATGCCTCCAGCAGTGAGG
LAMA3_R	TTTGTAATCCAGAGGTTGATTGACGCGTTTACTGGTCAGGACAACCATTCAGAC
LAMA3_qPCR_F	ACCCAGGCCAAGGACCTGAGG
LAMA3_qPCR_R	GTGTTGCCCGATTAACATTG
LAMA3A_qPCR_F	GTTCACAGCAGCAAAGGGTG
LAMA3A_qPCR_R	ACAAGCCTTTATGATCCCGATAG
LAMA3B_qPCR_F	AAGGAGCCAGCCTTTGTCAC
LAMA3B_qPCR_R	CTTGGGGAGGTCCTGCGTA
LAMB3_qPCR_F	GCAGCCTCACAACTACTACAG
LAMB3_qPCR_R	CCAGGTCTTACCGAAGTCTGA
LAMC2_qPCR_F	GGAGCTGGAGTTTGACACGA
LAMC2_qPCR_R	GAGCATGGAGCTGGAAGGTT
GAPDH_qPCR_F	TCTCCTCTGACTTCAACAGCGAC
GAPDH_qPCR_R	CCCTGTTGCTGTAGCCAAATTC
RPS13_qPCR_F	TCGGCTTTACCCTATCGACGCAG
RPS13_qPCR_R	ACGTACTTGTGCAACACCATGTGA
